# Disasters and Impacts in Appalachian Kentucky: A Behavioral Health Analysis

**DOI:** 10.13023/jah.0601.09

**Published:** 2024-09-01

**Authors:** Walter David Mathews, Joseph M. Clark, Amy S. Potts

**Affiliations:** Kentucky River Community Care; American Red Cross; Kentucky Department of Behavioral Health Developmental and Intellectual Disabilities

**Keywords:** Appalachia, behavioral health, disasters, emergency management, environmental stressors, integration of behavioral health, Kentucky, trauma

## Abstract

**Introduction:**

Major disasters continue to occur in Appalachian Kentucky with devastating consequences. A major disaster, defined by the Federal Emergency Management Agency (FEMA) as an event too large for a community to manage without outside help, involves emergency responders from the local, state, and federal disaster agencies, plus national volunteers.

**Purpose:**

This paper reports on recent disasters in eight southeast Kentucky counties, the changing nature of these disasters, and the behavioral health impact on the people affected.

**Methods:**

In this large-scale disaster survey in the Appalachian counties in Southeast Kentucky, over 3,500 people were asked about their recent disaster experiences in 2021 and 2022. The Disaster, Impact, and Screening Survey (DISS) was used to explore the respondent’s disaster history as a behavioral health client, general community member, or behavioral health professional, and how these views differed.

**Results:**

Respondents reported a higher rate of disaster experiences and requests for assistance than U.S. population surveys. Behavioral health clients and general community members disaster were not significantly coordinated but comparisons between behavioral health professionals clients were. Types of disasters and their impacts showed COVID pandemic caused the most widespread stressors such as school closings and missed work. Disasters such flooding caused the respondents property damage and homelessness Combining how widespread types of stressors and disaster severity ratings showed property damage, school closing, and home damage as the stressors with the greatest behavioral health impacts.

**Implications:**

Academic researchers and policymakers have expressed a desire to better integrate behavioral health services into the national emergency response system. To translate research into practice, health professionals need to better understand the disasters that have occurred in their service area, the types of impacts of those disasters, and how people have reacted. Local health providers should be involved in disaster preparedness, response, and long-term recovery as part of community resilience teams.

## INTRODUCTION

The Federal Emergency Management Agency (FEMA) reports that there have been 4,671 major disaster declarations nationally since 1950.^1^ Kentucky has had 87 major disasters, with the most frequent major disasters found in the eastern Appalachian portion of the state. *The New York Times* reported in 2018^2^ that Grayson, Kentucky, has been pummeled by nine major disasters in the last decade. Recent FEMA major disaster declarations for Kentucky have increased from one every few years to two or more per year.

Eastern Kentucky’s mountainous coalfield counties have steep terrain leading to narrow river valleys. Residents of the region have little choice for home building other than in these narrow valleys, which are prone to flooding. This challenging landscape has left little room for farming, other than small tobacco crops and family gardens. Along with flood disasters, other weather-related hazards include mudslides, wildfires, tornadoes, ice storms, and straight-line winds. The Eastern Kentucky flood disaster of July 27, 2022, left at least 40 dead.

FEMA disaster statistics tell just part of the story. The events that overwhelm a community’s ability to respond on its own are not limited to those tracked and declared by FEMA. Major disaster declarations focus on weather-related disasters and have only recently expanded to include biological or chemical disasters. Compared to those living elsewhere in the U.S., residents of Appalachia experience a broader range of disasters, including mining disasters,^3^,^4^ black lung disease,^5^ dam failures,^6^ polluted water,^7^ and school bus accidents.^8^ Ongoing disasters in the region include the opioid epidemic,^9^ and the lasting effects of the COVID-19 pandemic.^10^

Community and individual disaster resilience is dependent upon factors beyond preparedness, such as community stressors,^11^ rural isolation,^12^,^13^ and physical and mental health.^14^ Mao^15^ explores these social determinants to help guide public health policy in relation to disasters. Appalachian cultural traditions of self-reliance, spirituality, long-standing poverty, excessive chronic disease, and mistrust of outsiders^16^ can all contribute to the challenges of responding to disasters in the region.

The behavioral health factors caused by repeated traumatic disasters have received limited attention in the research literature from Appalachia.^17^–^18^ Despite the fact that in the past few decades, efforts have begun to integrate behavioral health into the emergency management system,^19^–^21^ there is still a need to add behavioral health services for Appalachian communities impacted by disasters.

In 2020, the Substance Abuse and Mental Health Services Administration (SAMHSA) and Kentucky Department of Behavioral Health (DBHDID) recognized the need to expand behavioral health services in recent major weather-related disaster areas. A 21-county Appalachian Kentucky region was selected for grant funding because of major weather-related disasters in 2019–2022. Included in this region is the eight-county area known as the Kentucky River Region (Breathitt, Knott, Lee, Leslie, Letcher, Owsley, Perry, and Wolfe Counties), where this study focused.

This paper reports on efforts to document not just the damage to homes and property from a series of disasters within the Kentucky River Region, but also the subsequent behavioral health stressors. Three questions guided the research:

- What are the types of disasters and impacts reported in these Appalachian Kentucky counties?
- Are there differences among the reported disasters and impacts on behavioral health clients v. others in the general community?
- Are there policy implications for state, local, and federal policymakers?

## METHODS

To screen for major disaster experiences and impacts, a screening survey was deployed. It identified the types of disasters that have occurred, their impact on the behavioral health of those affected by the disaster, and the interventions those individuals sought. To find disaster survivors, researchers created this Disaster Impacts Screening Survey (DISS), comprised of 10 items. The DISS instrument covered: (1) county of residence; (2) respondent age; (3) disaster(s) experience; (4) type of disaster(s); (5) when and where experienced; (6) weather or non-weather-related disasters; (7) type of impacts on respondent and family; (8) respondent group, (e.g., community member or seeking behavioral health services); (9) need for follow-up; and (10) contact information. Items were selected and evaluated using face validity and rational methods. The DISS was reviewed and approved by an internal review committee composed of the clinical staff of the agency and the DBHDID. People who wished to be contacted by agency staff gave personal identifying information that was not included in the survey analysis database.

### Data Collection

In January 2021, after direction by DBHDID, anyone seeking behavioral health services was screened using the DISS instrument either face-to-face or by telephone. Shortly thereafter, in March 2021, the area experienced another major flood disaster. Behavioral health staff in the impacted counties then went door to door with a DISS paper form to check on residents and ask them their status. Visitors and clients to behavioral health clinics were asked to complete the DISS in face-to-face interviews, at the clinic by self-report, or by calling in to a toll-free (1–800) crisis line for Kentucky River Community Care. Those who completed the survey received no compensation for doing so, although they could become eligible for SAMHSA-supported services with specified eligibility for disasters in 2019 or later. Additionally, community members were encouraged to go to an online website using JotForm^22^ by local TV news, radio stories, social media sites, community outreach events, and schools. Children under age 12 were surveyed using a parent or guardian as the intermediary; teenagers and older people, including older adults, provided self-reported data. These combined activities led to 3,999 surveys being completed. Anyone choosing to answer survey questions was initially included among the respondents. Based upon DBHDID requirements, the data collection period was January 2021 through April 2022.

### Data Analysis

The data collected—whether face-to-face, by telephone, or online—were analyzed by combining all surveys into the JotForm database. Subsequently, the data were downloaded into Excel format and entered into SPSS statistical analysis software.^23^ Data collection was anonymous unless the respondent asked for follow-up contact. After reviewing the data and removing incomplete or duplicate surveys, a total of 3,534 were used in the final data analysis. Surveys were considered incomplete if they lacked a combination of age, residence, and an answer of whether they had experienced a disaster. The DISS was also eliminated if the county of residence fell outside the ARC’s Appalachian boundary. Since each person was able to identify multiple disasters and impacts, the researchers rated each disaster by severity and the impacts by harmfulness.

## RESULTS

Table 1 shows the basic survey data. Tables 2–4 show additional results, which are discussed below. Among the 3,534 respondents remaining for analysis, 804 said they had experienced a disaster, representing 22.8% overall. This rate is slightly higher than previous studies of the U.S. overall population.^24^ Using the respondent category, over 58% of the respondents were general community members, and the remaining respondents were behavioral health clients and professional staff. While 545 (15.4%) requested follow-up contact from the behavioral health agency, upon follow-up, most initially said they just wanted help with property damages not covered by any other source, such as FEMA or insurance. Clinical staff interviewed all those requesting follow-up contacts to determine if behavioral health or other disaster services were needed. Each individual was connected to the most appropriate disaster service provider, such as emergency housing, food, and clothing services. Eventually, 740 of the 804 (92%) received behavioral health services when existing behavioral health clients were included.

### Community v. Clinical Samples

People identified themselves as community members, behavioral health clients, or other professionals such as behavioral health providers or educators. There was no significant difference between the community members’ ratings of impacts and those of the behavioral health clients (see Table 2). However, the ratings given by the behavioral health clients and the professional staff were highly correlated. Comparing the types of impacts between the behavioral health clients and the community sample found no significant differences. Although behavioral health clients and professional staff did agree more about the types of impacts experienced.

### Disaster Seriousness and Types of Impacts

Adapting methodologies discussed by Hasani^25^ and Caldera,^26^ the researchers weighted both the types of disasters and the impacts experienced by the respondents using a rational approach. For example, war, hurricanes, and earthquakes were rated as the most destructive (15) disasters; missing school was rated as the least destructive impact (2). Admittedly, Hasani and Caldera were looking at disasters from a worldwide disaster classification perspective rather than within Appalachia. This Kentucky-based research did not consider the tsunami or cyclones heavily weighted by Hasani, as these are historically rare in Appalachia. An impact score was given to each of the disaster and impact combinations by multiplying disaster frequency by the weights.


Impact Score=Disaster weight×frequency

By combining the types of disasters and the disaster impacts in the same table, one can assess the most salient combinations of these events in Appalachia; in Table 3 and Table 4, the frequency of the disaster-type combinations and their impacts in Appalachia becomes apparent. While the common types of disasters experienced in other parts of the country can be seen as having low frequency in this table, disasters more common in Appalachia are easily seen in the data. Experiences of stressors related to the COVID-19 pandemic, opioid epidemic, flooding, landslides, and mining accidents were most common in the sample, but very few mentioned the types of events common in coastal areas (hurricanes) or the Midwest (tornadoes). The most impactful disasters in this analysis are the combination of the COVID-19 pandemic and the resulting mandated school closings to prevent the spread of the disease (Table 3). These various impacts caused by COVID-19 were shown in all impact types, although flooding-related property damage came in a close second. The least impactful were heat waves and lives lost due to windstorms. The disaster types were rated from 1 (least harmful) to 15 (most harmful) based upon a combination of factors such as lives lost; damage to property, homes, and businesses; duration of the event; and warning prior to the event.

## DISCUSSION

Disaster impacts reported in this area of Appalachian Kentucky would undoubtedly change based on the millennial flood event of July 2022, which was not included here. Within the sample of 3,564 surveys, 22.8% reported having been involved in a disaster. The most common disaster in Appalachian Kentucky that leads to major disaster declarations is flooding. While in recent years the disasters described by the survey respondents included flooding, the impact of non-weather-related disasters, such the COVID-19 pandemic and the opioid epidemic, were among the top five Appalachian disasters. These non-weather-related disasters have major impacts, along with accidents not seen in other parts of the country. Comparing the Appalachian counties of Kentucky with other states or the rest of the nation presents a misleading view of what Appalachians experience as a disaster versus other areas—for example, mining accidents, water pollution, and school bus accidents.

Vulnerability to weather disasters, coupled with poverty and social vulnerability, does not usually reflect the impact of repeated trauma on health and well-being. Future researchers might include a broader range of disasters in considering community impacts, particularly in Appalachia. Weather disasters usually exclude other factors such as severe accidents, epidemics, or terrorist acts. Narrowly focusing on the impacts of weather disasters like property damage and home destruction may overlook the types of history and social factors that cause long-term behavioral health consequences. Repetitive trauma affects the development of children and the quality of life for older people.

One caveat to this study is that the research sample was a self-selected volunteer sample and not carefully designed to be representative of the region, although the size of the sample (3,564 of the 102,483 total area population) was large enough to reach a degree of reliability. The contemporaneous context of the disaster survey was during the period in which Western Kentucky had a tornado that took the lives of 80 people, making it the worst disaster in Kentucky’s history in terms of lives lost. Repeating the DISS in a multistate sampling of Appalachian counties may present a different picture of disasters. In fact, surveying any other region with a specific focus on behavioral health would be wise and helpful in planning behavioral health interventions.

The good news is that there are effective early interventions and treatments for children and adults. Disaster intervention researchers have reported success in treating disaster-related trauma.^27^–^29^ Widely accepted counseling techniques are being used during and immediately after a disaster in schools for suicide prevention using cognitive behavioral therapy techniques.^30^–^32^ In a new training manual for clinicians, Hamblen^33^ describes the evidence-based treatment approaches for clinicians to use in disaster response. This transdiagnostic approach promises to be a major step forward in disaster behavioral health treatment.

North^34^ describes the general principles for behavioral health inclusion in disaster response. Since academic and clinical training may not consider disaster response training, clinicians need to become proficient not only in the effective approaches to intervention but also in the cultural and diagnostic variations of intervention that work best in given disaster stages.^35^,^36^ The best way for behavioral health to become involved is through inclusion in the broader emergency management and medical response systems. This means that behavioral health professionals need to work before a disaster occurs on training and needs assessment so that when a disaster occurs, they are not starting from nothing when rapid response can be critical.^37^

Working within the existing emergency management framework and becoming knowledgeable about the incident command structure is necessary to become part of the overall response team. More Appalachian public health officials now see disaster preparedness as part of health programs as a step in the right direction. Others ask, as we prepare for disasters, “What are we preparing for?”^38^ Is the goal just survival during a major disaster? Programming that addresses disaster readiness challenges in prevention periods could save lives and costs. All Appalachian regions could have a disaster-trained behavioral health profession as a full-time member.

Bridging this readiness gap will prevent situations where people, communities, and systems survive the initial impact, but their resilience trajectories are vulnerable to the challenges of long-haul recovery. Palinkas^39^ describes a future when research evidence results in developing and implementing policies, programs, and practices. Future efforts will require the formation and maintenance of academic-community partnerships for the purpose of building resilience to these disaster impacts and providing targeted services to those most vulnerable. A new study by Rao^40^ on the characteristics of communities and individuals responding to disasters with resilience indicates that the traditionally disenfranchised or alienated are overlooked. The impoverished, minorities, and women, need to be better included in disaster preparedness activities. This study of an area of Appalachian disasters echoes those findings. However, this is preliminary analysis of extensive data on a complex issue. Appalachian disaster literature calls for more extensive research.

**SUMMARY BOX**

**What is already known about this topic?**

Academic researchers and policymakers have expressed a desire to better integrate behavioral health services into the national emergency response system. To translate research into practice, health professionals need to better understand the disasters that have occurred in their service area, the types of impacts of those disasters, and how people have reacted.

**What is added by this report?**

This paper reports on recent disasters in eight southeast Kentucky counties, the changing nature of these disasters, and the behavioral health impact on the people affected. In this large-scale disaster survey in the Appalachian counties in southeast Kentucky, over 3,500 people were asked about their recent disaster experiences in 2021 and 2022.

**What are the implications for future research?**

Future disaster research might consider the behavioral health impacts that go beyond the dollar amount of property damages and the number of fatalities. The trauma associated with disasters needs to be measured and behavioral health professionals included in the disaster assessments.

## Figures and Tables

**Table 1:** Survey Respondent Demographics (n = 3,534)

Appalachian County Resident	Yes		No		Total
	*n (%)*		*n (%)*		
	3,534 (100%)		0 (0%)		3,534 (100%)
**Age Group**	**0–12**	**13–17**	**18–30**	**31–50**	**51+**
	318 (9.0%)	402 (11.4%)	699 (19.8%)	1,298 (36.7%)	817 (23.1%)

**Ever Experience a Disaster**	**Yes**		**No**		**Do not Know**
	804 (22.8%)		2587 (73.2%)		143 (4.0%)
**When was Disaster**	**Before 2019**	**2019**	**2020**	**2021**	**No Answer**
	263 (7.4%)	77 (2.2%)	184 (5.2%)	292 (8.3%)	2,718 (76.9%)

**Respondent Category**	**Behavioral Health Client**	**Community Member**	**Behavioral Health Professional**	**Parent or Guardian**	**Other**
	1,235 (34.9%)	2,058 (58.2%)	189 (5.3%)	55 (1.4%)	3 (0.2%)

**Follow-up Requested**	**Yes**	**No**	**Missing/No Answer**		
	545 (15.4%)	2,768 (78.3%)	221 (6.3%)		

**Table 2:** Disaster Impacts by Respondent Category

Disaster Impacts	Respondent Category, n*		
	Community Member (CM)	Behavioral Health Client (BH)	Professional Staff (PS)
School Closings	178	263	28

Missed Work	202	142	15

Missed Doctor Appointments	55	91	5

Fear of Storms	130	40	14

Health Problems	139	162	42

Serious Psychological Distress	102	234	41

Alcohol/Drug Problems	47	257	35

Relapse	27	121	14

Serious Mental Illness	61	224	23

Property Damage	365	132	46

Home Damage	237	102	30

Lost Job	69	106	9

Food Shortage or Hunger	43	118	9

Homelessness	87	155	9

Family Separation	104	254	34

Family Member Killed or Injured	82	61	24

**Correlations** (with BH)			

CM	**– 0.062** *(not significant)*		

PS	**0.501** *(significant)*		

	**T-score = 0.251**	***t*** **-test significance**	
		*p* < 0.0001	

NOTES:

* Counts duplicated.

**Table 3:** Number of Respondents Indicating Having Experienced Various Disaster Impacts as a Stressor (duplicated count)

Disaster Impact	Disaster Type														
	War related	Mining Accident	Chemical Spill	Civil Unrest	Opioid Epidemic	Mud-or Landslide	Windstorm	Severe Storm	Heat Wave	Wildfires	Flooding	COVID	Tornado	Earthquake	Hurricane
School Closings	4	14	2	6	123	25	6	5	0	9	82	461	16	2	0

Missed Work	5	18	2	6	109	28	6	2	0	8	122	305	8	1	0

Missed Dr. Appointment	1	5	1	4	82	15	1	3	0	2	56	132	2	1	0

Fear of Storms	3	10	2	7	41	21	10	3	0	4	121	143	13	1	0

Health Problems	11	36	4	10	131	30	4	2	0	9	96	283	8	1	0

Serious Psych. Distress	4	20	5	22	172	34	4	4	0	9	102	311	13	0	1

Alcohol/Drug Problems	6	20	3	11	323	19	1	5	0	8	73	201	12	1	0

Relapse	5	10	2	6	149	7	0	2	0	4	38	90	5	0	0

Mental Illness	8	23	4	16	183	29	2	4	0	5	82	221	13	1	1

Property Damage	8	29	5	11	122	76	14	3	0	23	397	346	30	1	1

Home Damage	5	15	2	12	107	42	13	4	0	12	253	222	25	1	0

Lost Job	3	11	1	2	81	12	3	1	0	5	50	143	3	0	0

Hunger Food Shortage	2	2	2	2	86	10	2	3	0	3	50	135	3	0	0

Homelessness	3	13	1	6	136	19	1	0	0	9	97	146	8	0	1

Family Separation	6	20	1	11	246	25	2	5	0	4	97	282	10	0	1

Family Member Killed or Injured	6	44	3	7	90	18	0	1	0	5	47	122	7	1	0

**Table 4:** Intersection of Number of Disasters Experienced v. Weighted Stressor Impacts

		Disaster Weights (15 most serious)															Stressor impact scores by row	
		Mining Accident	Chemical Spill	Civil Unrest	Opioid Epidemic	Mud or Landslide	Windstorm	Severe Storm	Heat Wave	Wildfires	Flooding	COVID	Tornado	Earthquake	Hurricane	War Related		
Impact Stressor Weights ↓ (1 = least stressful; 15 = most)		2	3	4	5	6	7	8	9	10	11	12	13	14	15	15	Mean Score	Rank Order
**1**	School Closings	28	6	24	615	150	42	40	0	90	902	**5532**	208	28	0	60	515	2

**2**	Missed Work	36	6	24	545	168	42	16	0	80	1342	**3660**	104	14	0	75	407	6

**3**	Missed Doctor Appointments	10	3	16	410	90	7	24	0	20	616	1584	26	14	0	15	189	15

**4**	Fear of Storms	20	6	28	205	126	70	24	0	40	1331	1716	169	14	0	45	253	11

**5**	Health Problems	72	12	40	655	180	28	16	0	90	1056	**3396**	104	14	0	165	389	7

**6**	Psychological Distress	40	15	88	860	204	28	32	0	90	1122	**3732**	169	0	15	60	430	4

**7**	Alcohol/Drug Problems	40	9	44	1615	114	7	40	0	80	803	2412	156	14	0	90	362	8

**8**	Relapse	20	6	24	745	42	0	16	0	40	418	1080	65	0	0	75	169	16

**9**	Serious Mental Illness	46	12	64	915	174	14	32	0	50	902	**2652**	169	14	15	120	345	9

**10**	Property Damage	58	15	44	610	456	98	24	0	230	4367	**4152**	390	14	15	120	706	1

**11**	Home Damage	30	6	48	535	252	91	32	0	120	2783	2664	325	14	0	75	465	3

**12**	Lost Job	22	3	8	405	72	21	8	0	50	550	1716	39	0	0	45	196	12

**13**	Food Shortage or Hunger	4	6	8	430	60	14	24	0	30	550	1620	39	0	0	30	188	14

**14**	Homelessness	26	3	24	680	114	7	0	0	90	1067	1752	104	0	15	45	262	10

**15**	Family Separation	40	3	44	1230	150	14	40	0	40	1067	**3384**	130	0	15	90	416	5

**15**	Family Member Killed or Injured	88	9	28	450	108	0	8	0	50	517	1464	91	14	0	16	194	13

NOTE: Stressor Impact v. Disasters = Impact-severity Magnitude. Impact severity is a product of the type of disaster (including health epidemics, not just weather events) and stress it causes. The scores for impact severity reported by respondents in **bold** above reveal that COVID-related stress exceeded that of any weather-related disaster. Epidemics such as COVID-19 are a major behavioral health factor not usually accounted for in disaster declarations.
